# Assessment of neurologists’ knowledge regarding intravenous fibrinolytic therapy for acute stroke in Shanxi province in China

**DOI:** 10.1186/s12913-017-2300-6

**Published:** 2017-05-18

**Authors:** Liansheng Ma, Xiaoyuan Niu, Wei Zhang, Yalan Fang, Jie Wang

**Affiliations:** Department of Neurology, First Hospital, Shanxi Medical University, Taiyuan, 030000 People’s Republic of China

**Keywords:** Intravenous fibrinolysis, Ischemic stroke, Physicians’ knowledge, China

## Abstract

**Background:**

Limitations in physicians’ knowledge regarding fibrinolytic therapy for acute ischemic stroke may contribute to low rate of fibrinolytic therapy in China. Here physicians’ knowledge was surveyed on intravenous fibrinolytic therapy for acute ischemic stroke.

**Methods:**

Neurologists (*n* = 175) from 27 major general hospitals in Shanxi province, P. R. China, were invited to complete questionnaires regarding their basic knowledge of intravenous fibrinolytic therapy for acute ischemic stroke. The questionnaire contained 12 multiple-choice questions. One point was assigned for a correct answer and zero point for a false or unanswered question.

**Results:**

One hundred and thirty-one neurologists (74.9%) responded to the questionnaires. The mean accuracy rate of 12 questions was 54.9 ± 25.01% (range 0.8–96.2%). The mean total score for respondents was 6.59 ± 2.03 (range 2–11). More years of experience and higher academic degrees were independent factors related to the total scores (*P* = 0.000 and *P* = 0.004, respectively).

**Conclusions:**

The neurologists in this study were knowledge deficient in the area of intravenous fibrinolytic therapy for acute ischemic stroke. This partially accounts for the low rate of fibrinolytic therapy in China.

**Electronic supplementary material:**

The online version of this article (doi:10.1186/s12913-017-2300-6) contains supplementary material, which is available to authorized users.

## Background

Stroke is the second leading cause of death worldwide and the leading cause of death in China [1, 2]. The survivors of stroke have the highest ratio of severe overall disability among sufferers of chronic diseases [3]. It is a huge burden on Chinese medical insurance and the economy [4].

Ischemic stroke is the most common type of stroke in China [4]. It accounts for 43.7–78.9% of all strokes [2]. Intravenous fibrinolytic therapy for acute ischemic stroke can improve neurological outcomes along with reducing the potential degree of disability [5, 6]. It offers an effective treatment for stroke and the previous medical efforts chiefly focused on prevention of recurrence [7]. However, from September 2007 to August 2008, only 19.3% of patients with ischemic stroke presenting within 3 h received fibrinolytic therapy in 132 urban general hospitals in China [8]. Low education and old ages of patients chiefly accounted for the low rate of fibrinolytic therapy. However, this study did not include the factors related to the treating physicians in the multivariate logistic regression analysis. Some other studies regarding fibrinolytic therapy in developing countries mainly focused on the negative effects of financial constraint and insufficient infrastructure [9, 10]. Nevertheless, these factors do not work in China. Fibrinolytic therapy has been covered by healthcare insurance in China since 2008. Most major general hospitals could supply infrastructure for the therapy [8]. Therefore, it was hypothesized that physicians’ insufficient knowledge and understanding regarding fibrinolytic therapy contributes to the low rate of fibrinolytic therapy. For example, many Chinese physicians overemphasize the adverse effects associated with fibrinolytic therapy such as symptomatic intracerebral hemorrhage. With this in mind, the attitude of physicians can have a negative impact on patients or their families, who after consultation will likely refuse fibrinolytic therapy [8]. In this study, Chinese neurologists were surveyed regarding their knowledge of intravenous fibrinolytic therapy for acute ischemic stroke.

## Methods

This study was performed in Shanxi Province, P. R. China. Shanxi Province is a poorly developed area of China. The Development and Life Index (DLI), an index created by the National Bureau of Statistics of China, is used to assess an area’s average achievements in economic development, improvement of living standards, social development, ecological construction, and scientific innovation. In terms of DLI, Shanxi Province ranked 20th of the 32 provinces in China [11]. Shanxi Province consists of 11 regions. Twenty-seven major general hospitals are located in 10 of these regions.

There were two criteria for eligible neurologists: from the 27 major general hospitals and members of Shanxi Academy of Neurology. Between May and July 2014, questionnaires and invitations were mailed simultaneously to 175 eligible neurologists who were invited to attend Shanxi Academy of Neurology 2014 Biennial Meeting. The list of neurologists was obtained from Shanxi Academy of Neurology. Those who did not respond were considered to have dropped out. All participants consented before participation. This study was approved by the Ethics Committee of the First Hospital, Shanxi Medical University, Taiyuan, P. R. China.

The questionnaire included 12 multiple-choice questions (Additional file 1). The questions requested information regarding basic knowledge of intravenous fibrinolytic therapy for acute ischemic stroke. It included questions regarding examinations before fibrinolysis, inclusion criteria, exclusion criteria, doses of alteplase and the management of complications which were essential in the process of fibrinolysis. For convenience and to facilitate a higher response rate, the questionnaire was designed simply and did not include all aspects of knowledge regarding fibrinolysis.

The American Heart Association/American Stroke Association (AHA/ASA) guidelines were applied in this study [12]. The time window for treatment of acute ischemic stroke with intravenous fibrinolysis is 0 to 4.5 h. Imaging prior to intravenous fibrinolysis requires the use of non–contrast-enhanced computed tomography (NECT) or magnetic resonance imaging (MRI). In addition, blood glucose laboratory results are required before intravenous fibrinolysis. The dose of alteplase required for intravenous fibrinolysis is 0.9 mg/kg with a maximum dose of 90 mg. If hypodensity involves more than one third of the middle cerebral artery (MCA) territory on NECT, intravenous fibrinolysis should not be applied. Patients with prior ischemic stroke in the previous 3 months or previous intracranial hemorrhage should be excluded from intravenous fibrinolytic therapy. A National Institutes of Health Stroke Scale (NIHSS) score of greater than 25 is not an exclusion criterion for patients who can be treated within three hours. The NIHSS is a clinical assessment and research tool used for quantifying neurological injuries after stroke [13]. A high NIHSS score indicates severe symptoms. If the patient develops severe headache, acute hypertension, nausea or vomiting or has a worsening neurological examination, the infusion of alteplase should be discontinued.

If respondents did not write down an answer for a particular question, we presumed that they did not know the answer. The accuracy and unanswered rates of each question were calculated. The total score for each respondent was computed by assigning each of the 12 questions an appropriate individual score. A score of one was assigned for a correct answer and a score of zero for a false or unanswered question. Education was categorized into undergraduate and graduate degrees. Total scores and years of experience in the field of neurology were classified into two classes according to the median. Practice places were sorted into capital and non-capital cities. Univariate logistic regression analysis was used to identify the characteristics of respondents associated with the total scores, such as sex, education, years of experience and practice places. Multivariate logistic regression was performed to analyse the factors independently related to the total scores. All tests were two-tailed, and a *P* value of less than 0.05 was considered statistically significant. Data are presented as mean ± standard deviation unless otherwise specified. Data were analyzed using the Statistical Package for Social Sciences software, version 13.0 and Epicalc 2000.

## Results

One hundred and thirty-one neurologists (74.9%) from 27 hospitals responded to the questionnaires. All of them were certified physicians who practiced in departments of neurology. The chief characteristics of the study participants are shown in Table 1.

**Table 1:** The chief characteristics of the study participants

Characteristics	
n	131
Age, years	47.16 ± 8.41
Sex	
Male, n (%)	63 (48.09)
Female, n (%)	68 (51.91)
Education	
Undergraduate degree	70 (53.4)
Graduate degree	61 (46.6)
Years of experience	
≤ 23, n (%)	65 (49.62)
> 23, n (%)	66 (50.38)
Practice places	
Capital, n (%)	66 (50.38)
Non-capital, n (%)	65 (49.62)

The mean accuracy rate of 12 questions was 54.9 ± 25.01% (range 0.8–96.2%). The accuracy and unanswered rates are shown in Table 2. Question 3 regarding laboratory results required before fibrinolysis had the lowest accuracy rate (0.8%), while question 6 “whether thrombolyse is required if hypodensity on CT exceeds 1/3 MCA territory” had the highest accuracy rate (96.2%).

**Table 2:** Accuracy rate and rate of each unanswered question

Questions	Correct responses		Unanswered	
	n	% (95% CI)	n	%
1. Time window for intravenous fibrinolysis	85	64.9 (56.02–72.89)	1	0.8
2. Imaging results required before fibrinolysis	109	83.2 (75.45–88.95)	0	0
3. Laboratory results required before fibrinolysis	1	0.8 (0.05–4.87)	0	0
4. Dose of alteplase	87	66.4 (57.55–74.27)	3	2.3
5. Maximum dose of alteplase	67	51.1 (42.26–59.88)	4	3.1
6. Whether to thrombolyse if hypodensity > 1/3 MCA territory on CT	126	96.2 (90.89–98.6)	3	2.3
7. Whether to thrombolyse if patients had prior ischemic stroke within 2 months	84	64.1 (55.20–72.16)	2	1.5
8. Whether to thrombolyse if patients had previous intracranial hemorrhage	69	52.7 (43.82–61.42)	5	3.8
9. Whether to thrombolyse if NIHSS > 25 within 3 h from symptom onset	45	34.4 (26.46–43.27)	2	1.5
10. Whether to stop thrombolyse if patients develop skin, mucosa or gum bleeding	62	47.3 (38.58–56.18)	1	0.8
11. Whether to stop thrombolyse if patients develop severe vomiting	42	32.1 (24.37–40.9)	1	0.8
12. Whether to stop thrombolyse if patients develop worsening neurological examinations	86	65.6 (56.73–73.54)	1	0.8

*MCA* middle cerebral artery, *CT* computed tomography

The mean total score for respondents was 6.59 ± 2.03 (range 2–11). The distribution of total scores for respondents is listed in Table 3. None of the respondents attained full scores. 83.2% of respondents attained eight points or less and 46.6% attained six points or less.

**Table 3:** The distribution of total scores for respondents

Points	Respondents (n)	Percentage (%)
0	0	0
1	0	0
2	4	3.1
3	4	3.1
4	13	9.9
5	18	13.7
6	22	16.8
7	26	19.8
8	22	16.8
9	13	9.9
10	5	3.8
11	4	3.1
12	0	0

In univariate analysis, more years of experience and practicing in capital city significantly increased the total scores (*P* = 0.003 and *P* = 0.019, respectively; Table 4). Sex and higher degrees were found not to have a significant relation with total scores (*P* = 0.560 and *P* = 0.123, respectively). Multivariate analysis indicated that more years of experience and higher degrees were independent factors related to the total scores (*P* = 0.000 and *P* = 0.004, respectively; Table 4).

**Table 4:** Uniuariate and multivariate analysis for the association between total score categories and possible factors

Factors	Uniuariate results		Multivariate results	
	OR (95% CI)	*P* value	Adjusted OR (95% CI)	*P* value
Sex				
Male, n (%)	1	0.560	1	0.707
Female, n (%)	1.227 (0.617–2.441)		1.154 (0.545–2.443)	
Education				
Undergraduate degree	1	0.123	1	0.004
Graduate degree	1.729 (0.862–3.466)		3.608 (1.491–8.732)	
Years of experience				
≤ 23, n (%)	1	0.003	1	0.000
> 23, n (%)	3.000 (1.471–6.119)		5.317 (2.202–12.836)	
Practice places				
Capital, n (%)	1	0.019	1	0.116
Non-capital, n (%)	0.432 (0.214–0.872)		0.492 (0.203–1.192)	

*OR* odds ration, *CI* Confidence interval

## Discussion

Thrombolytic therapy is of proven and substantial benefit for select patients with acute cerebral ischemia. Nevertheless, currently there is a low rate of fibrinolytic therapy in China. Therefore, in this study, the objective was to explain the reasons for the low rate of fibrinolytic therapy from the perspective of physicians’ knowledge in Shanxi Province, P. R. China. A similar study has not been performed previously in China. The current study indicated that the neurologists were knowledge deficient in the area of intravenous fibrinolytic therapy for acute ischemic stroke. This partially accounts for the low rate of fibrinolytic therapy in China.

Here, it was showed that the accuracy rates of 12 questions pertaining to physicians’ knowledge in this area displayed a broad range (from 0.8 to 96.2%). The accuracy rates of half of all questions were lower than 60%. In general, these neurologists displayed the most optimal scores in the areas of CT imaging criteria of thrombolysis and necessary imaging before fibrinolysis. Good scores were also generated for questions relating to the time window of fibrinolysis and dose of alteplase. However, the majority of neurologists responded poorly to questions pertaining to side effects (such as severe vomiting and bleeding) associated with fibrinolysis and decisions regarding how to take action to stop the side effects or progression of stroke. The mean total score for respondents was very low. Most respondents (83.2%) scored eight points or less. None of the tested respondents answered all questions correctly. However, in the fibrinolytic procedure, any mistake could lead to undesirable consequence. For example, loosened inclusion criteria may cause cerebral hematoma. Only those who get full scores may handle the procedure correctly. The situation could not satisfy the demand of healthcare. Our findings suggest that neurologists in Shanxi province displayed limited knowledge regarding the criteria of intravenous fibrinolytic therapy for acute ischemic stroke.

Medicine develops rapidly. Physicians need to update their professional knowledge continuously in order to adapt to new situations associated with stroke-care. There are currently numerous academic conferences that facilitate continuing healthcare education in China; however, educational training is not compulsory and the supervision system is defective. This is likely to be one of the main reasons behind the poor performance associated with fibrinolytic therapy. Furthermore, due to the risk of adverse effects such as symptomatic intracerebral hemorrhage, and the potential of undesirable consequences associated with fibrinolytic procedures, many patients and/or their families delay or refuse fibrinolytic therapy [8]. The effects of poor knowledge of neurologists and misunderstanding of public may leaded to the low fibrinolytic uptake rate in China.

In this study, all the respondents were from major hospitals of Shanxi Province, P. R. China. It has previously been demonstrated that the level of physicians’ knowledge in China can be affected by the level of hospitals that they come from [14]. Indeed, it is likely that if physicians from county or community hospitals were included in this study, the results would be significantly worse. However, the process of intravenous fibrinolysis should be conducted near to patient residence, due to the fact that long distance transport wastes time and can lead to further neuronal death and missing the therapeutic window.

There are many ways to improve the quality of stroke-care. For instance, shortening the waiting times for a CT scans, or reducing the time prior to intravenous alteplase administration, along with increasing the rates of intravenous alteplase use can all potentially improve stroke care quality [15, 16]. With this in mind, it is critical for neurologists to be familiar with the guidelines associated with the early management of patients with acute ischemia. This would likely facilitate clinicians in limiting the morbidity and mortality associated with stroke. Greater knowledge in this area would also help to improve stroke prevention, care within the first few hours of acute stroke onset and even prognosis.

This study has several limitations. Firstly, for convenience and to facilitate a higher response rate, the questionnaire was designed simply and did not include all aspects of knowledge regarding fibrinolysis. Even if respondents attained maximal scores in this questionnaire, they might not manage optimally in practice. Secondly, the subjects were from major hospitals of Shanxi Province and did not represent physicians throughout the whole country. It would have been more optimal to obtain substantial data through cooperation with hospitals of different provinces. Thirdly, the respondents were members of the Shanxi Academy of Neurology and were not randomly selected.

## Conclusions

Neurologists in this study had poor knowledge regarding intravenous fibrinolytic therapy for acute ischemic stroke. This situation is unsatisfactory and may partially account for the low fibrinolytic rate in China. Physicians’ continuing education needs to be facilitated and improved. Because respondents with lower degrees or less year of experience tended to have lower total scores, these physicians should have more chances. Compulsory relevant training and suitable assessment should be carried out to ensure that physicians have the necessary knowledge and skills. This does not exclusively pertain to early stroke management through fibrinolysis but also pertains to the medical system in China as a whole. We have reported the information to the Shanxi Stroke Association and more relevant course will be offered to physicians. Public education is also urgently needed to increase the low fibrinolytic rate.

## Additional file

Additional file 1: Survey of neurologists’ knowledge on intravenous fibrinolytic therapy for acute stroke. (DOC 28 kb)

## Abbreviations

AHA/ASA: American Heart Association/American Stroke Association
CI: Confidence interval
CT: Computed tomography
DLI: Development and Life Index
MCA: Middle cerebral artery
MRI: Magnetic resonance imaging
NECT: Non–contrast-enhanced computed tomography
NIHSS: National Institutes of Health Stroke Scale
OR: Odds ration

## References

[1] Lozano R, Naghavi M, Foreman K, Lim S, Shibuya K, Aboyans V (2012). Global and regional mortality from 235 causes of death for 20 age groups in 1990 and 2010: A systematic analysis for the global burden of disease study 2010. Lancet.

[2] Liu M, Wu B, Wang WZ, Lee LM, Zhang SH, Kong LZ (2007). Stroke in china: Epidemiology, prevention, and management strategies. Lancet Neurol.

[3] Adamson J, Beswick A, Ebrahim S (2004). Is stroke the most common cause of disability?. J Stroke Cerebrovasc Dis.

[4] Liu L, Wang D, Wong KS, Wang Y (2011). Stroke and stroke care in china: Huge burden, significant workload, and a national priority. Stroke.

[5] The National Institute of Neurological Disorders and Stroke rt-PA Stroke Study Group (1995). Tissue plasminogen activator for acute ischemic stroke. N Engl J Med.

[6] Hacke W, Kaste M, Bluhmki E, Brozman M, Davalos A, Guidetti D (2008). Thrombolysis with alteplase 3 to 4.5 hours after acute ischemic stroke. N Engl J Med.

[7] Fugate JE, Giraldo EA, Rabinstein AA (2010). Thrombolysis for cerebral ischemia. Front Neurol.

[8] Wang Y, Liao X, Zhao X, Wang DZ, Wang C, Nguyen-Huynh MN (2011). Using recombinant tissue plasminogen activator to treat acute ischemic stroke in china: Analysis of the results from the chinese national stroke registry (cnsr). Stroke.

[9] Ghandehari K (2011). Barriers of thrombolysis therapy in developing countries. Stroke Res Treat.

[10] Ghandehari K, Zahed AP, Taheri M, Abbasi M, Gorjestani S, Ahmadi AM (2009). Estimation of iranian stroke patients eligible for intravenous thrombolysis with tpa. Int J Stroke.

[11] Reginal development and life index reports of 2011. Beijing: National Bureau of Statistics of China; 2014. http://www.stats.gov.cn/tjzs/tjsj/tjcb/dysj/201404/t20140417_540868.html. Accessed 21 Aug 2014.

[12] Jauch EC, Saver JL, Adams HP, Bruno A, Connors JJ, Demaerschalk BM (2013). Guidelines for the early management of patients with acute ischemic stroke: A guideline for healthcare professionals from the american heart association/american stroke association. Stroke.

[13] Goldstein LB, Bertels C, Davis JN (1989). Interrater reliability of the nih stroke scale. Arch Neurol.

[14] Ji L, Newman J, Lu J, Cai X (2014). Understanding the standard of care in the treatment of type 2 diabetes in china: Results from a national survey. Chin Med J (Engl).

[15] Rose KM, Rosamond WD, Huston SL, Murphy CV, Tegeler CH (2008). Predictors of time from hospital arrival to initial brain-imaging among suspected stroke patients: The north carolina collaborative stroke registry. Stroke.

[16] Sung SF, Ong CT, Wu CS, Hsu YC, Su YH (2010). Increased use of thrombolytic therapy and shortening of in-hospital delays following acute ischemic stroke: Experience on the establishment of a primary stroke center at a community hospital. Acta Neurol Taiwanica.

