# Supplementary material for: Assessment of neurologists’ knowledge regarding intravenous fibrinolytic therapy for acute stroke in Shanxi province in China
Source: BMC Health Serv Res. 2017 May 18;17:358. doi: 10.1186/s12913-017-2300-6 (PMC5437417; doi:10.1186/s12913-017-2300-6)
Supplement: Additional file 1: — Survey of neurologists’ knowledge on intravenous fibrinolytic therapy for acute stroke. (DOC 28 kb) [file 12913_2017_2300_MOESM1_ESM.doc]

Appendix

Survey of neurologists’ knowledge on intravenous fibrinolytic therapy for acute stroke

1. Which is (are) within time window of intravenous fibrinolysis for acute stroke?

A. 0 - 3 hours B. 0 - 4.5 hours C. 0 - 6 hours D. 0 - 12 hours

2. Which imaging result (s) is (are) required before fibrinolysis?

A. CT B. MRI C. CTA D. MRA

3. Which laboratory result (s) is (are) required before fibrinolysis?

A. Blood glucose B. Coagulation tests C. Liver and kidney function tests

D. Blood routine E. Blood troponin and myoglobin

4. How to calculate the dose of alteplase when stroke patients receive intravenous fibrinolytic therapy?

A. < 0.6 mg/kg B. 0.6 - 0.9 mg/kg C. 0.9 mg/kg

5. Which is the maximum dose of alteplase when stroke patients receive intravenous fibrinolytic therapy?

A. 90 mg B. 99 mg C. 110 mg

6. Should intravenous fibrinolytic therapy be performed if hypodensity involves more than one third of the MCA territory on CT?

A. Yes B. No

7. Should intravenous fibrinolytic therapy be performed if patients have prior ischemic stroke within 3 months?

A. Yes B. No

8. Should intravenous fibrinolytic therapy be performed if patients have history of previous intracranial hemorrhage?

A. Yes B. No

9. Should intravenous fibrinolytic therapy be performed if patients have severe stroke (NIHSS > 25) within 3 hours from symptom onset?

A. Yes B. No

10. Should intravenous fibrinolytic therapy be discontinued if patients develop skin, mucosa or gum bleeding?

A. Yes B. No

11. Should intravenous fibrinolytic therapy be discontinued if patients develop severe vomiting？

A. Yes B. No

12. Should intravenous fibrinolytic therapy be discontinued if patients develop worsening neurological examinations?

A. Yes B. No

MCA, middle cerebral artery; CT, computed tomography; MRI, magnetic resonance imaging; CTA, Computed tomography angiography; MRA, Magnetic Resonance Angiography.
